# Case report of cryptogenic multifocal ulcerous stenosing enteritis (CMUSE): a rare disease may contribute to endoscopy-capsule retention in the small intestine

**DOI:** 10.1186/s12876-019-0962-8

**Published:** 2019-04-03

**Authors:** En-Wei Tao, Tian-Hui Zou, Yong-Feng Wang, Jie-Ting Tang, Ying-Xuan Chen, Qin-Yan Gao

**Affiliations:** Division of Gastroenterology and Hepatology, Shanghai Institute of Digestive Disease, State Key Laboratory for Oncogenes and Related Genes, Key Laboratory of Gastroenterology & Hepatology, Ministry of Health, Ren-Ji Hospital, Shanghai Jiao-Tong University School of Medicine, 145 Middle Shandong Road, Shanghai, 200001 China

**Keywords:** Cryptogenic multifocal ulcerous stenosing enteritis, Capsule retention, Diagnosis, Anemia

## Abstract

**Background:**

CMUSE is a rare disease whose diagnosis remains difficult because the lesion is confined to the small bowel.

**Case presentation:**

Here, we present a case of 43-year-old female patient suffered chronic abdominal pain for 20 years, and finally diagnosed with CMUSE. Capsule endoscopy was performed when general endoscopic investigation failed to find the lesion, but the capsule was stranded in the small intestine. Moreover, capsule retention results in acute intestinal obstruction. Thus, surgery was performed and CMUSE was confirmed. The patient was recovered after partial small intestine resection.

**Conclusions:**

Capsule retention occurred in nearly 60% of patients with CMUSE. Capsule endoscopy should be avoided when the patient is suspected of CMUSE, especially with severe anemia and radiologic finding in the ileum.

## Background

Cryptogenic multifocal ulcerous stenosing enteritis (CMUSE) is a rare disease with unknown etiology and pathophysiology. This disease is an independent entity characterized by chronic and intermittent bouts of moderate ileus resulting from multiple short stenoses of the small bowel with shallow ulcers [1–3]. It has been reported in both adults and children but were mainly diagnosed during adulthood with a mean age of approximate 40 years [2, 4–6]. The clinical characteristics of CMUSE include abdominal pain and iron-deficiency anemia, moreover, the location of ulcerative strictures was usually located in the ileum [4, 5, 7]. The diagnosis of CMUSE remains difficult because of the unspecific clinical manifestations and vague radiologic findings such as the presence or absence of strictures and superficial ulcers in abdominal computed tomography (CT) or small bowel series (SBS). Thus, small intestine wireless capsule endoscopy (CE) seems to be the best non-invasion inspection of CMUSE. However, capsule retention in the bowel is becoming a common complication of CMUSE should not be neglected.

## Case presentation

A 43-year-old woman with remittent abdominal pain, dizziness, and fatigue for 20 years was admitted to our hospital in May 2018. She was also suffered from iron deficiency anemia since her adolescence. There were positive results of stool occult blood test but no evidence of bleeding in gastroscopy and colonoscopy during the progression of the disease. On examination, she had lower limbers edema and mild abdominal tenderness around the umbilicus. Vital signs were within normal range.

**Fig. 1:**
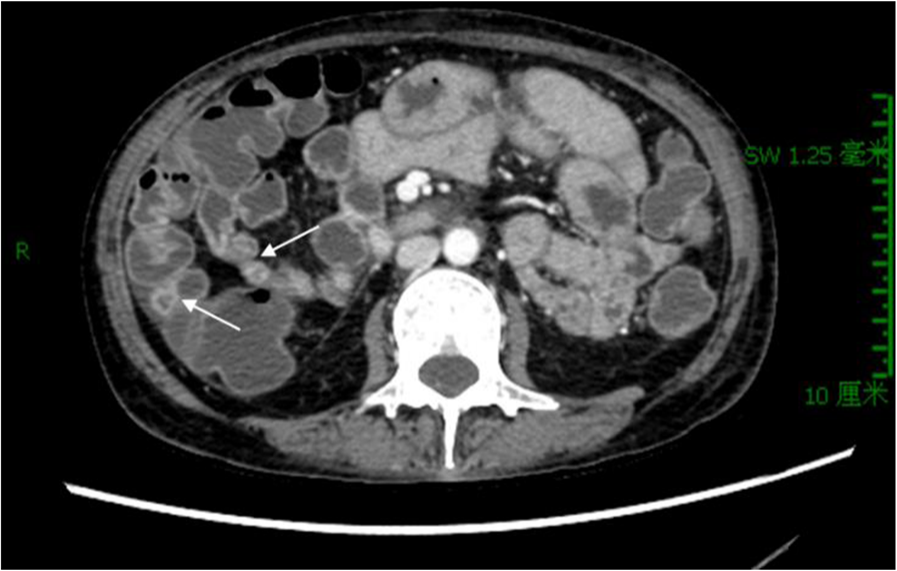
Radiograph of the abdominal computed tomography (CT). The findings show segmental bowel walls are thickened (arrows)

**Fig. 2:**
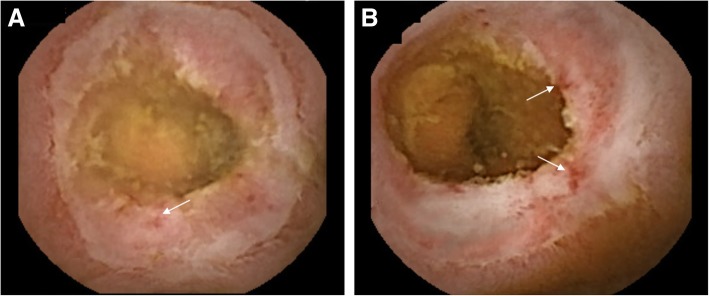
Capsule endoscopy (CE) findings. **a**&**b**: superficial and circular ulcers in the small intestine, and there are small bleeding spots (arrows) on the surface of the ulcers

**Fig. 3:**
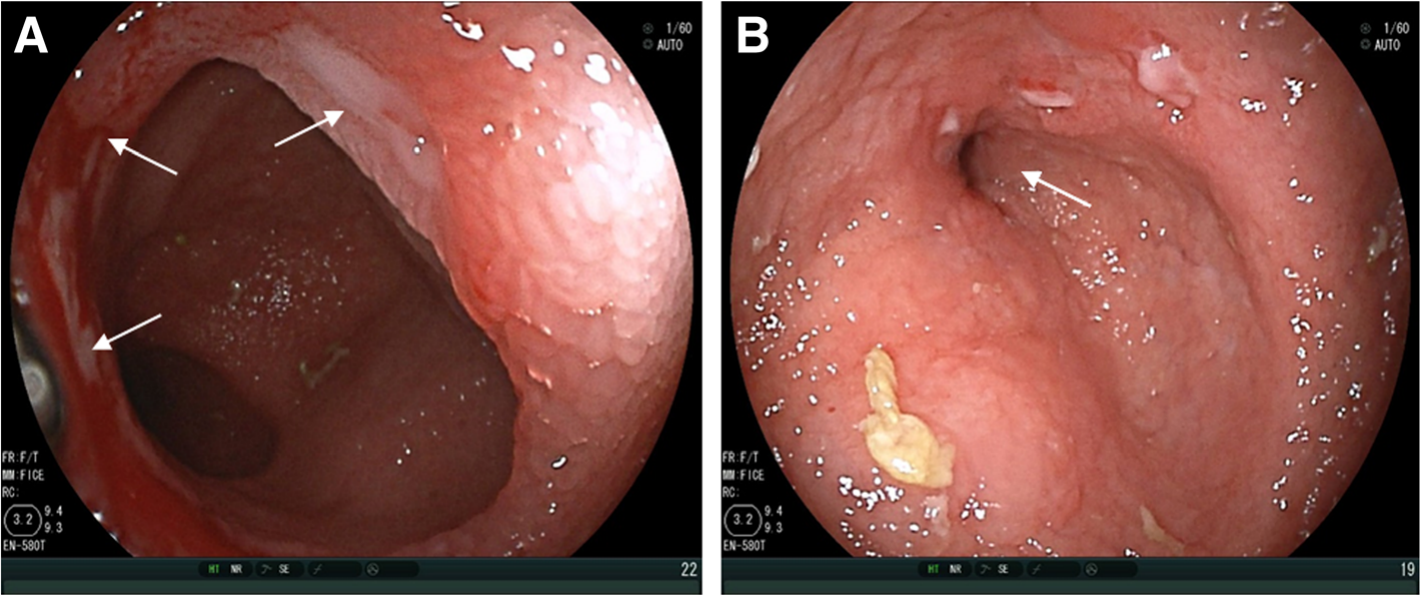
Double balloon endoscopy (DBE) findings. **a**: shallow ulcers (arrows) surround the intestinal wall. **b**: severe stenosis (arrow) results in endoscopy termination

Her past medical history included appendectomy, oophorocystectomy, and caesarean section. She denied NSAIDs taking. Laboratory examination confirmed iron deficiency anemia (hemoglobin level 86 g/L, normal range: 130–175 g/L) and hypoalbuminemia level (14.7 g/L, normal range: 35–55 g/L), C-reaction protein (30.95 mg/L, normal range: 0.08–7.6 mg/L) was elevated. In addition, the fecal occult blood test was positive. Other test results, such as renal and liver functions, autoimmune antibodies, T-spot were within normal range. Meanwhile, colonoscopy and gastroscopy showed normal results. However, abdominal CT scan revealed segmentally thickened small intestinal walls but no mention of stenosis (Fig. 1). Due to the presence of a metal intrauterine device, magnetic resonance (MR) enterography was prohibited. Therefore, CE was used for further inspection. The result found multiple circular ulcerations, and stenoses (Fig. 2). Double balloon endoscopy (DBE) was performed for capsule retained which showed multiple ulcerative stenoses (Fig. 3) but failed to find out the retained capsule. Biopsy report suggested nonspecific moderate chronic inflammation, and villi became widen and shorten. The culture of bacteria or acid-fast stain for tuberculosis were negative. Based on the results above, CMUSE was diagnosed temporarily. The patient received a tentative treatment of methylprednisolone (40 mg/day) and parenteral nutrition and the retained capsule was waiting for discharge by itself.

**Fig. 4:**
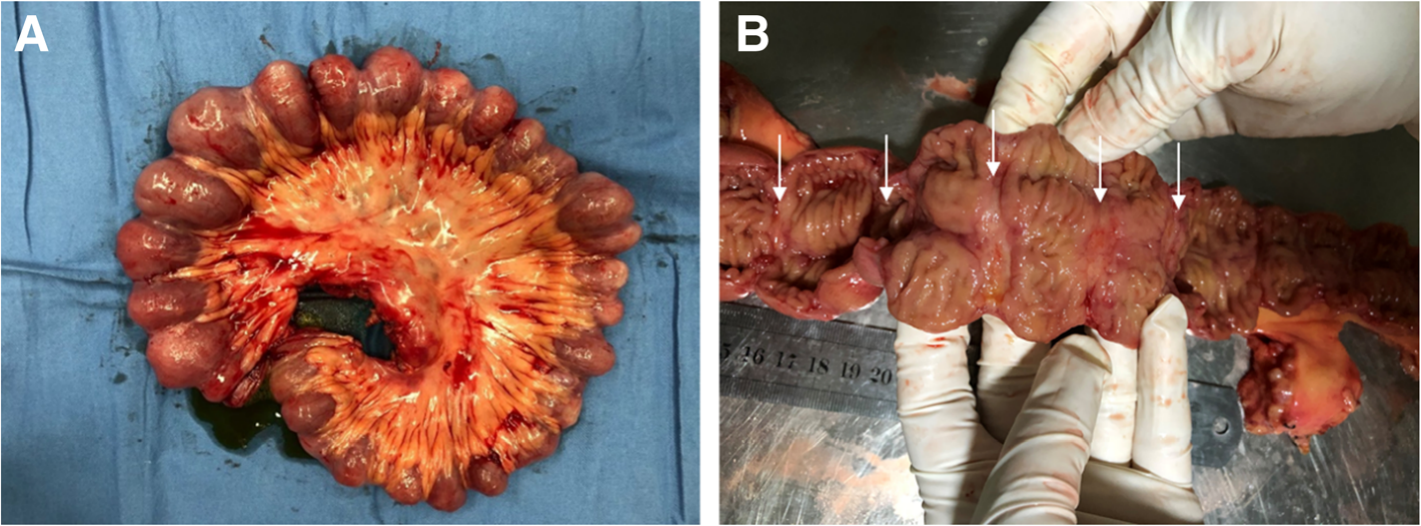
Resection specimen of the small bowel. **a**: a section of small intestine about 60 cm with approximate 23 strictures every 1.5–3.5 cm. **b**: multiple and regularly arranged circular ulcers (arrows)

**Fig. 5:**
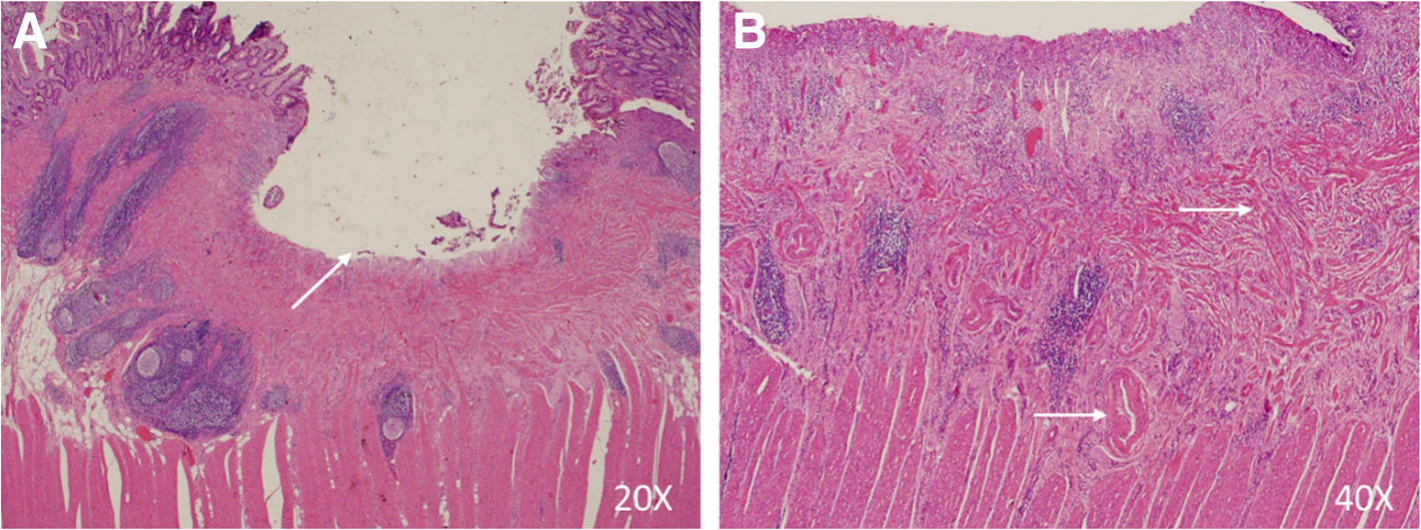
Histological findings on the tissue specimen (H&E staining). **a**: the ulcer (arrow) is confined to the mucosal layer. **b**: muscularis mucosae (arrow, above) and small blood vessels (arrow, below) were markedly thickened and disordered

Unfortunately, exploratory laparotomy was performed because of acute intestinal obstruction which may be induced by retained capsule after 2 weeks. During the operation, multiple segmental stenoses of the small intestinal were observed and the endoscopy-capsule was found in the ileum. A section of the small intestine, about 60 cm in length, was removed. There were approximately 26 circumferential superficial ulcers with strictures and the minimal distance between two strictures was about 1.5–3.5 cm (Fig. 4). Postoperative pathology showed superficial ulcerations which limited to the mucosal and submucosal layers (Fig. 5) without granulomas, lymphadenopathy or vasculitis. Finally, CMUSE was diagnosed and she was recovered well after the surgery. Oral steroids were suggested to the patient, but she refused.

## Discussion and conclusions

Since Debray et al. [1] reported the first case of CMUSE in 1964, this rare disease has been found in the worldwide region. and Matsumoto et al. [8] call this distinctive entity chronic nonspecific multiple ulcers (CNSU) of the small intestine. However, CNSU is now considered as a different entity called chronic enteropathy associated with SLCO2A1 gene (CEAS) [9]. Moreover, neuromuscular and vascular hamartoma (NMVH) is supposed to be the same disease of CMUSE [10]. The etiology of CMUSE is still unclear, but several hypotheses such as immunopathological pathogenesis [3], excessive formation of fibrous tissue and disturbance of collagen degradation [3, 11], vasculopathy induced by partial C2 deficiency [2, 12], recessive mutations in the PLA2G4A [13] genes had been suspected.

CMUSE is a chronic and recurrent disease. The most common symptoms include abdominal pain, anemia, and gastrointestinal bleeding [4, 5]. Diagnosis of this disease remains difficult due to the lack of specific characteristics and the lesion mainly located in the small bowel. Differential diagnosis of CMUSE includes Crohn’s disease (CD) [14, 15], NSAIDs-induced enteropathy [16], tuberculous enteritis and other chronic infections of the small intestine [17–19].

In order to directly observe the lesions in the small intestine, both CE and DBE are viable [20–22]. Generally, capsule endoscopy, serve as non-invasive detection, is preferred to use before balloon endoscopy. However, the capsule retention rate of CMUSE patients is extremely high which should not be neglected. After reviewing pieces of literature of CMUSE and some cases in China, 22 patients with CMUSE were performed CE and the presence of capsule retention is also clearly described. Surprisingly, 13 patients underwent capsule retention [3, 5, 10, 14, 17, 23–30]. It means that nearly 60% of patients with CMUSE who receives CE will contribute to retention in the small intestine. Capsule retention has been reported in approximately 1.4% of CE procedures [31]. Even in the patient with known CD, the incidence rate is only about 13% [32].

Capsule retention is a potential disadvantage of intestinal obstruction which may require endoscopic or surgical retrieval of the capsule [33, 34]. Moreover, some capsules will disintegrate in the small bowel [3, 27]. However, the reason why some patients with CMUSE are more likely to undergo capsule retention remains unknown. For further study, a total of 12 patients with CMUSE were finally included in the analysis. Among them, 7 patients have undergone capsule retention and 5 patients have not (Table 1). Based on their clinical characteristics, we found severe anemia and the location of lesion are the main causes of capsule retention. Notably, almost all the patients with capsule retention had some radiologic findings, but unfortunately, those findings were inconspicuous. Therefore, we suggest that patient who is suspected of CMUSE with severe anemia and radiologic findings in the ileum, should avoid using CE which may lead to capsule retention. For these patients, DBE is recommended for further inspection. As the development of patency capsule, we suggest those patients with CMUSE and do not have severe anemia and radiologic findings in the ileum take patency capsule before CE to minimize risk of retention.

**Table 1:**
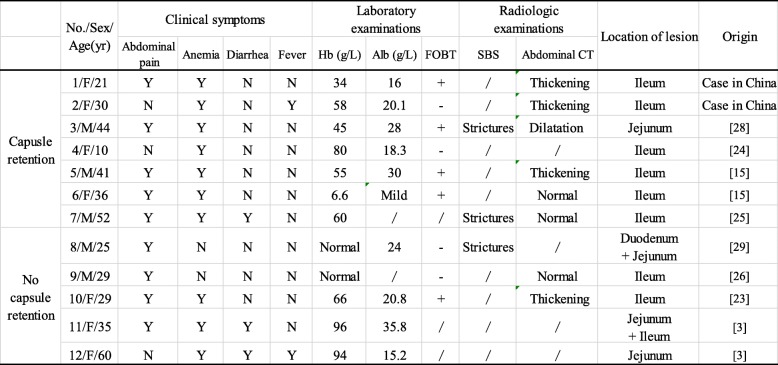
Clinical characteristics of the patients diagnosed with CMUSE

/: Not done or clearly described; *FOBT* Fecal occult blood test, *SBS* Small bowel series, *CT* Computed tomography

As for treatment, the most effective is immunotherapy [2, 3] and surgery. However, the recurrence rate of CMUSE is high [2, 5], and most patients develop corticosteroid dependence. Intriguingly, infliximab (anti-TNF-α therapy) was reported useful in CMUSE [26]. Moreover, gene mutations are closely related to this disease [35], genetic therapy may be effective for this rare disease in the future.

## Abbreviations

CD: Crohn’s disease
CE: Capsule endoscopy
CEAS: Chronic enteropathy associated with SLCO2A1 gene
CMUSE: Cryptogenic multifocal ulcerous stenosing enteritis
CNSU: Chronic nonspecific multiple ulcers
CT: Computed tomography
DBE: Double balloon endoscopy
MR: Magnetic resonance
NMVH: Neuromuscular and vascular hamartoma
SBS: Small bowel series
