# Correction to: Case report of cryptogenic multifocal ulcerous stenosing enteritis (CMUSE): a rare disease may contribute to endoscopy-capsule retention in the small intestine

**DOI:** 10.1186/s12876-019-0983-3

**Published:** 2019-04-29

**Authors:** En-Wei Tao, Tian-Hui Zou, Yong-Feng Wang, Jie-Ting Tang, Ying-Xuan Chen, Qin-Yan Gao

**Affiliations:** Division of Gastroenterology and Hepatology, Shanghai Institute of Digestive Disease, State Key Laboratory for Oncogenes and Related Genes, Key Laboratory of Gastroenterology & Hepatology, Ministry of Health, Ren-Ji Hospital, Shanghai Jiao-Tong University School of Medicine, 145 Middle Shandong Road, Shanghai, 200001 China

**Correction to: BMC Gastroenterol**

**https://doi.org/10.1186/s12876-019-0962-8**

Following publication of the original article [1], the author reported the wrong version of Table 1 has been published. The word of 'Capsule' was mistakenly written as 'Capusle'. The correct version of the table can be found below:

**Table 1:** Clinical characteristics of the patients diagnosed with CMUSE

No./Sex/ Age(yr)	Clinical symptoms				Laboratory examinations			Radiologic examinations		Location of lesion	Origin
	Abdominal pain	Anemia	Diarrhea	Fever	Hb (g/L)	Alb (g/L)	FOBT	SBS	Abdominal CT		
Capsule retention											
1/F/21	Y	Y	N	N	34	16	+	/	Thickening	Ileum	Case in China
2/F/30	N	Y	N	Y	58	20.1	-	/	Thickening	Ileum	Case in China
3/M/44	Y	Y	N	N	45	28	+	Strictures	Dilatation	Jejunum	[28]
4/F/10	N	Y	N	N	80	18.3	-	/	/	Ileum	[24]
5/M/41	Y	Y	N	N	55	30	+	/	Thickening	Ileum	[15]
6/F/36	Y	Y	N	N	6.6	Mild	+	/	Normal	Ileum	[15]
7/M/52	Y	Y	Y	N	60	/	/	Strictures	Normal	Ileum	[25]
No capsule retention											
8/M/25	Y	N	N	N	Normal	24	-	Strictures	/	Duodenum + Jejunum	[29]
9/M/29	Y	N	N	N	Normal	/	-	/	Normal	Ileum	[26]
10/F/29	Y	Y	N	N	66	20.8	+	/	Thickening	Ileum	[23]
11/F/35	Y	Y	Y	N	96	35.8	/	/	/	Jejunum + Ileum	[3]
12/F/60	N	Y	Y	Y	94	15.2	/	/	/	Jejunum	[3]

/: Not done or clearly described; *FOBT* Fecal occult blood test, *SBS* Small bowel series, *CT* Computed tomography

The original article has been corrected as well.
